# Ectopic expression of *AtPAD4* broadens resistance of soybean to soybean cyst and root-knot nematodes

**DOI:** 10.1186/1471-2229-13-67

**Published:** 2013-04-25

**Authors:** Reham M Youssef, Margaret H MacDonald, Eric P Brewer, Gary R Bauchan, Kyung-Hwan Kim, Benjamin F Matthews

**Affiliations:** 1 USDA-ARS, Soybean Genomic and Improvement Laboratory, 10300 Baltimore Ave, Bldg 006, Beltsville, MD, 20705, USA; 2 Cell and Genetics Division, National Institute of Agricultural Biotechnology, Rural Development Administration, Suwon, 441-100, South Korea; 3 Plant Protection Department, Faculty of Agriculture, Fayoum University, Fayoum, Egypt

**Keywords:** Arabidopsis, Phytoalexin-deficient4, AtPAD4, Soybean, Nematodes, Salicylic acid, Plant defense

## Abstract

**Background:**

The gene encoding *PAD4* (PHYTOALEXIN-DEFICIENT4) is required in *Arabidopsis* for expression of several genes involved in the defense response to *Pseudomonas syringae* pv. *maculicola. AtPAD4* (*Arabidopsis thaliana PAD4*) encodes a lipase-like protein that plays a regulatory role mediating salicylic acid signaling.

**Results:**

We expressed the gene encoding *AtPAD4* in soybean roots of composite plants to test the ability of *AtPAD4* to deter plant parasitic nematode development. The transformed roots were challenged with two different plant parasitic nematode genera represented by soybean cyst nematode (SCN; *Heterodera glycines*) and root-knot nematode (RKN; *Meloidogyne incognita*). Expression of *AtPAD4* in soybean roots decreased the number of mature SCN females 35 days after inoculation by 68 percent. Similarly, soybean roots expressing *AtPAD4* exhibited 77 percent fewer galls when challenged with RKN.

**Conclusions:**

Our experiments show that *AtPAD4* can be used in an economically important crop, soybean, to provide a measure of resistance to two different genera of nematodes.

## Background

Inducible defense responses are activated when plants respond to pathogen attack [1]. The gene-for-gene defense response is a strong form of plant resistance against pathogens. This type of resistance is turned on when the plants have a specific resistance (*R*) gene that recognizes the product of a corresponding pathogen gene known as the avirulence (*avr*) gene. This interaction between an *R*-gene and an *avr*-gene triggers the hypersensitive response (HR) and rapid expression of defense responses that result in programmed cell death within 24 h of infection [2]. Another type of defense response occurs after attack by virulent pathogens that do not have an *avr*-gene recognized by the plant. In this case, the plant responds more slowly than in gene-for-gene resistance, allowing the pathogen to multiply. Resistance to virulent pathogens can occur through a phenomenon called systemic acquired resistance (SAR) [3], which occurs after the hypersensitive response. SAR reduces symptoms produced by a variety of pathogens, but not by all pathogens [4]. Salicylic acid (SA) plays a central signaling role in both gene-for-gene resistance and SAR. This role for SA was proven by construction of transgenic plants expressing a bacterial salicylate hydroxylase gene (*nahG*) that converts SA to catechol [5]. During gene-for-gene resistance or infection with virulence pathogens, the *nahG* plants failed to express pathogenesis related (*PR)* genes, and their susceptibility to both virulent and avirulent pathogens was greatly enhanced [5,6].

Mutants of the model plant *Arabidopsis thaliana* have been used for studying SA-dependent regulation of plant defense responses. Production of certain defense signals is controlled by the *Arabidopsis PAD4* (*AtPAD4*) gene. Plants carrying *PAD4* mutations displayed reduced levels of SA, decreased expression of the defense gene *PR1*, and reduced synthesis of the indole derivative, camalexin, after infection with a virulent strain of *Pseudomonas syringae*[7-10]. In contrast, the role of *PAD4* in defense against the green peach aphid *Myzus persicae* Sulzer is independent of SA and camalexin [10,11]. Reported that *PAD4* encodes a nucleo-cytoplasmic protein which has similarity to triacyl glycerol lipases and other esterases. In defense signaling, *PAD4* acts in conjunction with the *EDS1* gene (ENHANCED DISEASE SUSCEPTIBILITY1), which encodes a structurally related protein also found in the nucleus and cytoplasm [12,13]. EDS1 is required for accumulation of PAD4 protein [14]. EDS1 also interacts with another lipase-like protein, SAG101 (SENESCENCE-ASSOCIATED GENE101), which accumulates in the nucleus [13]. The occurrence of EDS1-PAD4 and EDS1-SAG101 complexes inside plant cells suggests that EDS1 works as an adaptor for both PAD4 and SAG101 in defense signaling [13].

Although *PAD4* has been extensively studied in *Arabidopsis*, less is known about its role in conferring resistance to nematodes, and it is not known if *AtPAD4* can function in economically important crops, such as soybean, to provide resistance to nematodes. The soybean cyst nematode (SCN; *Heterodera glycines*) and the root-knot nematode (RKN; *Melidogyne incognita*) are obligatory plant parasites that are responsible for more than 100 billion U. S. dollars in yield losses annually of economic crops worldwide [15]. Both nematode species establish complex feeding sites within their host plants. At infection, the pre-parasitic second stage juveniles (J2) penetrate the roots and migrate towards the vascular cylinder where they induce the growth of a multinuclear feeding site, termed a ‘syncytium’ produced by SCN and a ‘giant cell’ produced by RKN [16].

The SCN life cycle, which can be completed in about 30 days under optimum conditions, includes six stages: the egg, four juvenile stages, and the adult [17]. The only stage to infect plant roots is the J2, which is motile and typically penetrates the host root and migrates to the vascular cylinder while secreting cell-wall degrading enzymes [18-21]. Once there, the nematode injects proteins into a host cell through its stylet, inducing formation of the syncytium [22-26]. Many physiological and morphological changes occur during formation of the syncytium: surrounding cell walls partially dissolve, nuclei enlarge, the density of organelles in the cytoplasm increases, and there is an accumulation of endoplasmic reticulum [24,27,28]. Initiation and formation of the syncytium is a complicated process requiring an unknown host signal transduction pathway triggered by secretions from the nematode esophageal glands [18,20]. After the feeding site is initiated, the J2 molts to the J3 and J4 stages before finally developing into a female or male adult. The female remains sedentary at the feeding site while the mature male becomes mobile in the root to fertilize the female. The female extracts nourishment from the syncytium to support the production of several hundred eggs, most of which stay inside the female’s body, while others are excreted as a gelatinous mass into the soil. After the female dies, the body remains intact and hardens into a tough leathery sac known as a cyst. Eggs and larvae can survive in the cyst body for several years until they are stimulated to hatch in the soil under favorable conditions [18,25,26].

The life cycle of RKN varies from three weeks to several months depending on environmental factors such as temperature, moisture, and availability of a suitable host [29]. The infective second stage juveniles (J2) penetrate the roots of the host plant using the piercing action of their stylets. Once inside, the nematode releases esophageal secretions which induce the formation of a multinucleate feeding cell. The J2 becomes sedentary, feeds, and undergoes three molts (J3, J4, adult). Occasionally vermiform males develop and migrate out of the roots, while females remain sedentary, feeding and producing eggs in a gelatinous matrix. Embryogenesis begins inside the egg, and J_2_ individuals hatch after the first molt [30].

In this work, we demonstrate that overexpression of the *Arabidopsis* gene *AtPAD4* in transgenic soybean roots of composite plants can confer resistance to both SCN and RKN.

## Results

### *Agrobacterium* transformation of soybean roots with red fluorescent protein *(RFP)*

**Figure 1:**
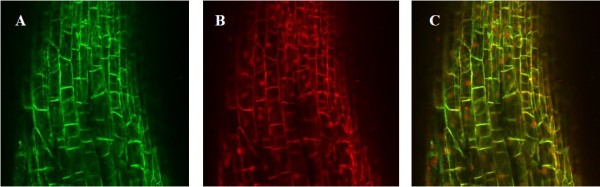
**Confirmation of the effectiveness of the plant overexpression vector pRAP15. A**, *eGFP* [green fluorescence], **B**, *RFP* [red fluorescence], and **C**, *RFP* and *eGFP* together; magnification 25X.

The *RFP* gene was cloned into the pRAP15 vector and expressed in soybean roots to confirm the overexpression functionality of the pRAP15 vector (Figure 1). Transformed roots were identified by the presence of green fluorescent protein (eGFP) throughout the root (Figure 1A). Strong red fluorescence demonstrated that the figwort mosaic virus subgenomic transcript (FMV) promoter was successful in expressing the *RFP* gene in the transformed soybean roots. Strong green fluorescence throughout the root demonstrated that the *rol*D promoter was successful for driving the *eGFP* gene (Figure 1B). When the images were overlapped, the red and green fluorescence were co-localized (Figure 1C). The magnification was 25X.

### *Agrobacterium* transformation of soybean roots with *AtPAD4*

**Figure 2:**
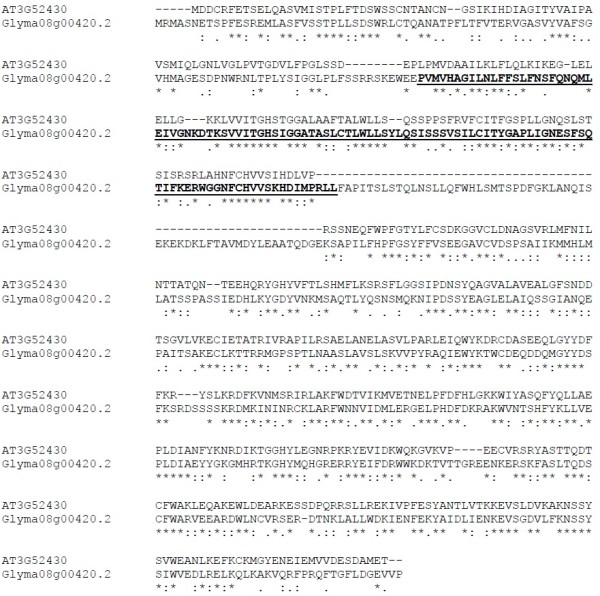
**Protein sequence alignment of the coding region of the *PAD4 *gene from Arabidopis and soybean.** *, identical amino acid residues aligning in both sequences, **:,** different but highly conserved (very similar) amino acids aligning in both sequences, **.,** different amino acids that are somewhat similar aligning in both sequences, **-,** this column of the alignment contains dissimilar amino acids or gaps, Bold, underlined letters identify the lipase 3 motif.

We cloned the *Arabidopsis PAD4* (*AtPAD4*) gene into pRAP15 for overexpression in transgenic soybean roots of composite plants. The amino acid sequence of *AtPAD4* (AT3G52430) is moderately conserved with the closest soybean homolog Glyma08g00420.2 (Figure 2). *AtPAD4* (AT3G52430.1) shares 41.8% amino acids identity with GmPAD4 (Glyma08g00420.2). Both proteins possess a lipase 3 motif conserved throughout numerous proteins.

Of 100 soybean plants subjected to root transformation with the *AtPAD4* gene, 55% showed evidence of transformation 28 days after planting as shown by eGFP fluorescence. The transformation efficiency for the empty pRAP15 control plants was 74%. After partial trimming of the untransformed roots and an additional 14 days of growth, all untransformed roots were removed and the remaining roots displaying strong eGFP fluorescence were inoculated with RKN or SCN for assay.

### Molecular analysis of putative transgenic plants

**Figure 3:**
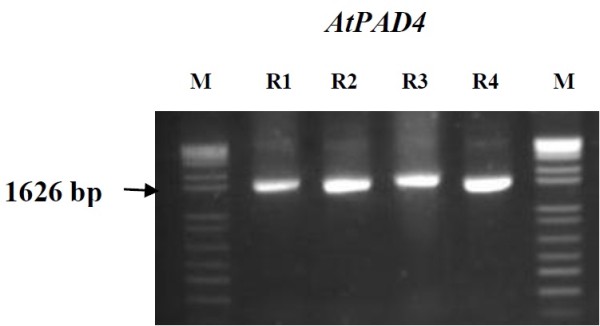
**PCR showing the presence of the *AtPAD4 *insert in transgenic lines.** The size of the amplicon is indicated by an arrow, M, is molecular weight standard, R1-4, represents PCR amplicons from DNA extracted from individual roots.

The insertion of the *AtPAD4* gene fragments in transgenic soybean plants was detected by PCR (Figure 3) using gene specific primers (Table 1). The 1626 bp fragment was amplified with the gene specific primers. Four plants were tested and all were shown to contain transgenic DNAs. No amplification was detected in untransformed control roots and control roots transformed with empty pRAP15.

**Table 1:** Primers used in PCR amplification and sequencing

**Name**	**Sequences [5′-3′]**
***AtPAD4*-F**	CACCAGCCAAGAAGATACATA
***AtPAD4*-R**	TTC GAT TTG CTA TTA GTC CTA
**FMV-F**	GGAGCCCTCCAGCTTCAAAG
***eGFP*-F**	ATCGATGAATTTGTTCGTGAACTATTAGTTGCGG
***eGFP*-R**	ATCGATGCATGCCTGCAGGTCACTGGATTTTG
***RFP*-F**	CACCTGATGGCCTCCTCCGAG
***RFP*-R**	TTAGGCGGTGGAGTG G

### qRT-PCR to determine the expression of *AtPAD4* gene in soybean roots

**Figure 4:**
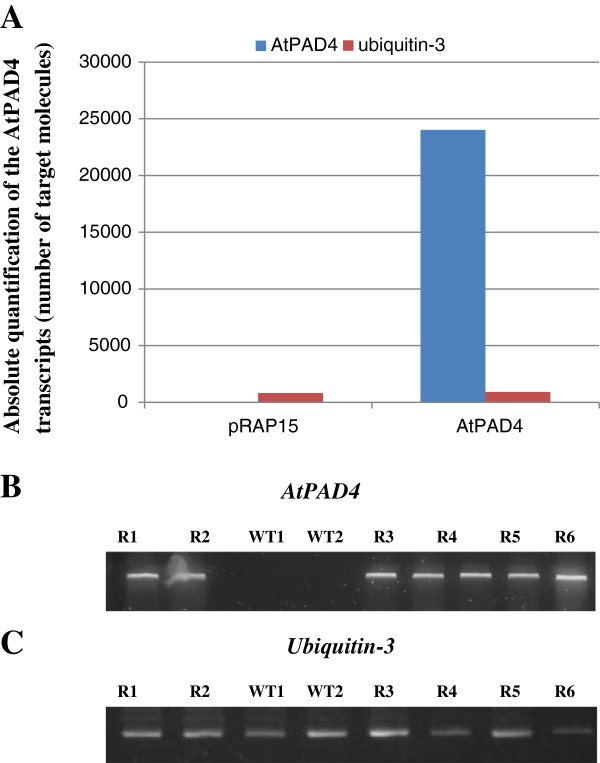
**Quantitative real time-PCR results. A**, the mRNA transcript level of the *AtPAD4* gene in the overexpressing roots and empty vector (control) and the non-target *Ubiquitin-3* gene transcripts. The x-axis represents the experiment type. The y-axis represents the absolute quantification of the mRNA transcript of different genes (number of target molecules), **B**, Showing the presence of the *AtPAD4* insert in transgenic line, **C**, Showing the presence of the non-target *Ubiquitin-3* gene transcripts, M, is molecular weight standard, R1-6, represents PCR amplicons from RNA extracted from individual roots.

Roots expressing eGFP were further analyzed to determine the abundance of *AtPAD4* gene transcripts by qRT-PCR using gene specific primers (Table 2). The absolute quantification of the transcripts (number of target molecules) was calculated using the sigmoidal method described by [31]. *AtPAD4* transcripts in the overexpressing roots were abundant, while the control roots displayed no detectable to the *AtPAD4* (Figure 4A). The number of transcripts of *AtPAD4* in the roots transformed with the *AtPAD4* construct was calculated to be 24030 molecules. Although transcripts of *AtPAD4* were not detectable in the control roots containing empty vector (Figure 4B), transcripts of the housekeeping gene encoding ubiquitin-3 were similar in all samples (Figure 4C).

**Table 2:** Primers used in qRT-PCR amplification and sequencing

**Name**	**Sequences [5′-3′]**
***AtPAD4*-F**	CACCAGCCAAGAAGATACATA
***AtPAD4*-R**	TTCGATTTGCTATTAGTCCTA
***Ubiquitin-3*-F**	GTGTAATGTTGGATGTGTTCCC
***Ubiquitin-3*-R**	ACACAATTGAGTTCAACACAAACCG
***GmPR1-F***	GCATCATGAATTTAGCCAACG
***GmPR1-R***	TTCCAGGTGACCAAGCAAGT
***GmPAD4-F***	GGGAAGGGGATGCACACAACCAAGG
***GmPAD4-R***	GTTGGCCATTCCATCCTTCCACCACCT
***GmEDS1-F***	CGTGAAGAGGCTTGTGCTAGCAGGGGTATG
***GmEDS1-R***	CAATGTCTAGAGGCTCCACAAGGCGGCG

**Figure 5:**
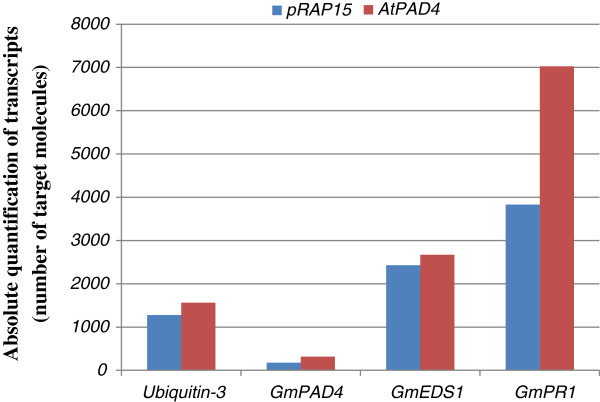
**Quantitative real time-PCR results showing the mRNA transcript level of the *GmPAD4, GmEDS1 *and *GmPR1 *gene.** In the overexpressing roots and empty vector (control). Also, The non-target *Ubiquitin-3* gene transcripts. The x-axis represents the experiment type. The y-axis represents the absolute quantification of the mRNA transcript of different genes (number of target molecules).

In addition to measuring transcript levels of *AtPAD4,* we also used qRT-PCR to determine the number of transcripts of three defense-related genes, *GmPAD4*; *GmEDS1* and *GmPR1* (Figure 5). The number of transcripts of *GmPAD4* in roots overexpressing *AtPAD4* were almost double the number found in control roots. In the same roots, the number of transcripts of *GmEDS1* did not change significantly between *AtPAD4-*overexpressing roots and control roots. However, the number of transcripts of *GmPR1* in *AtPAD4-*overexpressing roots was almost double that found in control roots containing empty vector.

### Effect of *AtPAD4* overexpression in soybean roots resistance

#### Resistance to soybean cyst nematode

**Figure 6:**
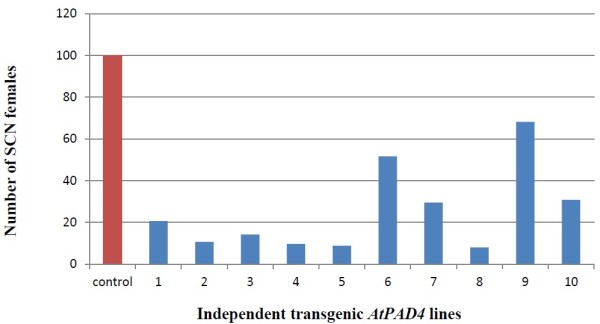
**Bars for the *AtPAD4 *lines showing the reduction in the number of SCN females**. In the PAD4-transformed roots of individual plants divided by the fresh weight of the root compared to the mean of the number of cysts found on roots of control plants transformed with empty pRAP15 divided by the mean of the fresh weight of the roots, pRAP15,control transformed with the empty pRAP15 vector, *AtPAD4*, transformed with the *AtPAD4* construct.

**Figure 7:**
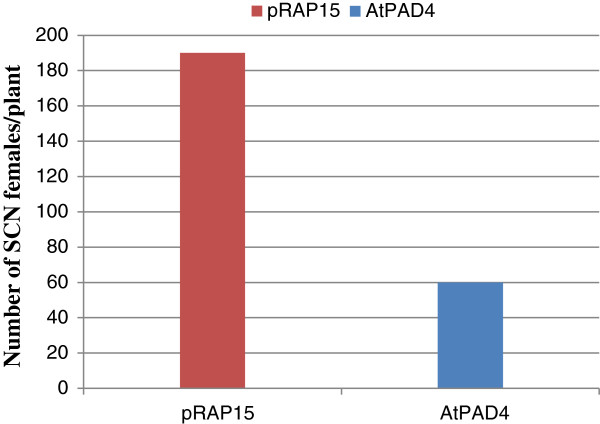
**Bars represent the mean number of mature SCN females per plant**. pRAP15, control transformed with the empty pRAP15 vector, *AtPAD4*, transformed with the *AtPAD4* constructs.

The effect of overexpression of *AtPAD4* in roots of the susceptible soybean cultivar ‘Williams82’ on the development of SCN females was examined by counting the number of mature SCN females on *AtPAD4-*overexpressing and control roots 35 days after inoculation (dai) (Figure 6). There was a 68% reduction in the mean number of SCN females per plant on *AtPAD4*-overexpressing roots as compared to the pRAP15 control (Figure 7). When expressed as number of SCN females per gram of root wet weight, the reduction was 76% in the *AtPAD4*-overexpressing plants. These differences are considered to be statistically significant (P<0.0001), indicating that the expression of *AtPAD4* in soybean roots interrupted the development of SCN females (Table 3).

**Table 3:** **Number of mature SCN females collected 35 dai from roots overexpressing *AtPAD4 *and from control roots**

**Treatment**	**No. of females/ plant**	**Root wet weight [g]**	**No. of females/g root wet weight**
**NC**	178 ± 77	5.1 ± 1.4	35 ± 19
**pRAP15**	190 ± 60	7.3 ± 1.6	26 ± 6.9
***AtPAD4***	60 ± 41	9.5 ± 3.5	6.3 ± 5.3

Mean ± standard deviation [N = 10].

NC = nontransformed control; pRAP15 = empty pRAP15 control.

#### Resistance to root knot nematode

**Figure 8:**
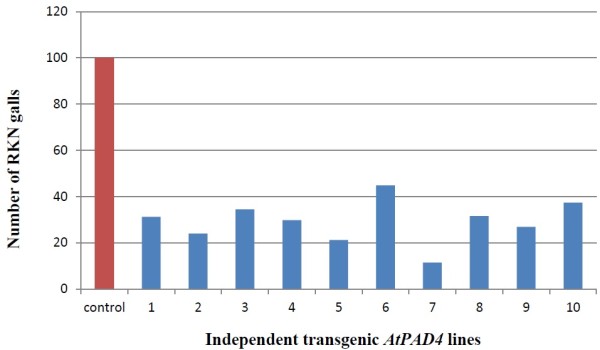
**Bars for the *AtPAD4 *lines showing the reduction in the number of RKN galls.** In the PAD4-transformed roots of individual plants divided by the fresh weight of the root compared to the mean of the number of cysts found on roots of control plants transformed with empty pRAP15 divided by the mean of the fresh weight of the roots. pRAP15, control transformed with the empty pRAP15 vector, *AtPAD4*, transformed with the *AtPAD4* constructs.

**Figure 9:**
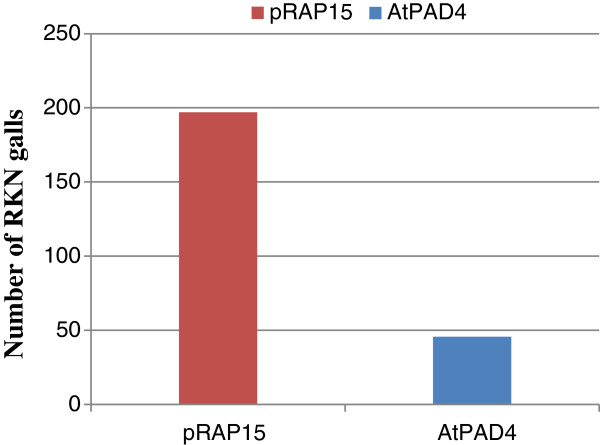
**Bars represent the mean number of in the Number of RKN galls.** pRAP15, control transformed with the empty pRAP15 vector, *AtPAD4*, transformed with the *AtPAD4* constructs.

The effect of overexpression of *AtPAD4* in roots of the susceptible soybean cultivar ‘Williams82’ on the development of RKN galls was examined by counting the number of galls on *AtPAD4-*overexpressing and control roots 35 dai (Figure 8). Under blue light, galls were easily identified as solid, thick green regions on the transformed roots. The mean number of RKN galls per plant was 77% lower on *AtPAD4*-overexpressing plants, compared to the pRAP15 control (Figure 9). When expressed as number of RKN galls per gram of root wet weight, the reduction was 72% in the *AtPAD4*-overexpressing plants. These differences are considered extremely statistically significant (P<0.0001) and indicate that the *AtPAD4* gene interrupted RKN development (Table 4).

**Table 4:** **Number of RKN galls counted 35 dai on roots overexpressing *AtPAD4 *and on control roots**

**Treatment**	**No. of galls/ plant**	**root wet weight [g]**	**No. of galls/ g root wet weight**
**NC**	173 ± 63	8.0 ± 3.8	22 ± 10
**pRAP15**	198 ± 51	8.1 ± 1.8	24 ± 3.1
***AtPAD4***	46 ± 18	6.6 ± 2.2	6.9 ± 2.2

Mean ± standard deviation [N = 10].

NC = nontransformed control; pRAP15 = empty pRAP15 control.

The size of RKN galls and of RKN nematodes within roots was determined 35 dai by measuring the area of their profiles using the Leica Microsystem software version 5.0 for the laser capture microscope. The profile area of RKN galls in roots transformed with *AtPAD4* was 86% smaller than that of RKN galls on control roots (Table 5). Similarly, the profile area of RKN nematodes was 66% smaller in *AtPAD4* roots. Thus there were many more immature nematodes in *AtPAD4* roots than in control roots. We also observed fewer egg masses on the *AtPAD4* roots.

**Table 5:** S**ize of RKN galls and immature females as measured by the areas of their profiles**

**Treatment**	**Gall profile area [mm**^**2**^**]**	**Nematode profile area [mm**^**2**^**]**
**pRAP15**	1.57 ± 0.63	0.083 ± 0.03
***AtPAD4***	0.23 ± 0.15	0.028 ± 0.007

Mean ± standard deviation [N = 10].

pRAP15 = empty pRAP15 control.

## Discussion

### Plant pathogen interaction

Sedentary endoparasitic nematodes comprise a large group of plant pathogens that infect and parasitize the roots of their hosts. The interaction between these nematodes and their hosts is highly complex, and their obligate root-parasitic nature has proven to be a hindrance to the molecular characterization of these pathosystems, including the targeted exploration of plant defense responses during nematode parasitism. As a consequence, compared with foliar bacterial, viral, and fungal pathogens, there is a considerable lack of knowledge regarding which defense signaling genes or pathways are effective against plant-parasitic nematodes during a compatible interaction.

Effective plant defense against pests and pathogens involves recognition and activation of appropriate defenses. Similar underlying mechanisms are likely to control this fundamental process in all flowering plants [32]. Therefore, structural and functional analysis of genes involved in plant defense in a model species such as *Arabidopsis thaliana* (L.) Heynh, can facilitate the identification of structural and functional orthologs and their role in disease resistance pathways in other plant species [1,8].

Natural plant populations and breeding populations of crop plants show qualitative and quantitative phenotypic variation for resistance to pests and pathogens. Qualitative resistance is characterized by two distinct phenotype classes, resistant and susceptible, and follows Mendelian inheritance. It is this type of single gene- or resistance (R) gene-mediated resistance that has been most thoroughly studied in the context of plant-pathogen recognition and defense signaling [33-36]. In contrast, quantitative resistance is characterized by continuous phenotypic variation ranging from high susceptibility to high resistance among the recombinant individuals within a progeny. Such resistance is controlled by more than one gene and can be strongly influenced by environmental factors. Resistance to SCN is controlled by several resistance (*Rhg,* resistance to *Heterodera glycines*) genes [37-40], and soybean cultivars can display a range of reactions to SCN encompassing highly susceptible to resistant depending upon the SCN population used for testing and the complement of *Rhg* genes within the genome of the cultivar. The resistance genes appear to work in a SCN population-specific or race-specific manner and most contribute only a small, additive amount to resistance [37,38].

### *Arabidopsis*-pathogen interactions

The genetic dissection of *Arabidopsis*-pathogen interactions revealed great insights into plant defense and various defense signaling pathways. Our knowledge of *R* gene-activated defenses, as well as regulators of salicylic acid (SA), jasmonic acid (JA), and ethylene (ET) dependent response pathways has expanded greatly in the last two decades [33,41]. Analyses of *A. thaliana* mutants that perturb various aspects of SA-mediated signal transduction has revealed that SA is an inhibitor of cyst nematode parasitism during a compatible interaction [42]. Mutants unable to synthesize or accumulate SA (*sid2-1*, *pad4-1*, and *nahG*) showed a consistently increased susceptibility phenotype to *H. schachtii*. The pretreatment of wild-type plants with SA significantly decreased their susceptibility to the nematode while simultaneously inducing PR-1 gene expression in both roots and shoots. Taken together, these data strongly suggest that SA mediated signaling plays a significant role in limiting nematode parasitism during a compatible interaction. [43]showed that application of SA to tomato plants prior to inoculation with root-knot nematode alleviated root galling; however, this effect of SA was believed to be nematicidal in nature due to the high concentrations of SA that were used in the experiment. [10,44] showed that *PAD4* modulates camalexin synthesis and SA synthesis and signaling in Arabidopsis defense against pathogens.

Although, there have been extensive studies on the defense response of *Arabidopsis* to fungal and bacterial pathogens (1–6), little of this work in *Arabidopsis* has been directly translated to economically important food crops, such as soybean, and particularly in respect to plant parasitic nematodes. The *Arabidopsis* lipase-like protein PHYTOALEXIN DEFICIENT4 (PAD4) was identified by several genetic studies as essential component for plant immunity against virulent pathogens for promoting the defense signaling hormone salicylic acid (SA) and the phytoalexin camalexin [8-10,33]. Our experiments show that *AtPAD4*can be used in an economically important crop, soybean, to provide a measure of resistance to two different genera of nematodes. In *Arabidopsis,* the *PAD4* gene functions upstream of the defense responses triggered by SA [45]. PAD4 can work in combination with EDS1 to trigger aspects of the defense response, but EDS1 can also interact with SAG101 independent of PAD4 [45]. In our experiments, overexpression of *AtPAD4* in soybean roots did not greatly influence levels of transcripts of *GmEDS1,* but there was an increase in *Gm PR1* transcripts. *PR1* transcription is responsive to increased SA levels and is downstream of SA [46-48]. Overexpression expression of *AtPAD4* in transgenic *Arabidopsis* conferred resistance to green peach aphid [49]. This resistance did not require EDS1. Our data, extend understanding of *AtPAD4* by showing that this *Arabidopsis* gene can be overexpressed in an economically important crop to confer resistance to two distinct genera of nematodes.

## Conclusions

Here, we demonstrated that the overexpression of *AtPAD4* in roots of *G. max* confers resistance to two different genera of nematodes. It decreases the number of mature female SCN cysts and decreased the number of galls formed by RKN in the susceptible soybean cultivar ‘Williams82’. Moreover, the size of RKN galls and nematodes in *AtPAD4*-expressing roots was significantly reduced and fewer egg masses were present, confirming that the ectopic overexpression *AtPAD4* in soybean roots disrupted the RKN life cycle. This work provides a basis for unraveling the potential role of defense signaling genes in quantitative disease resistance in this major crop species, and it demonstrates that an *Arabidopsis* gene can confer resistance in an important field crop to two genera of nematodes having worldwide importance.

## Methods

### Nematode procurement

SCN (*H. glycines*) females were harvested from soybean (*G. max*) roots 2–4 months after inoculation. The females were purified by sucrose flotation .and then crushed gently to release the eggs. The eggs were sterilized by 0.5 percent sodium hypochlorite solution for 1.5 min then washed with sterile water and placed in a small plastic tray with 120 mL sterile water and 1.2 mL sterile 300 mM ZnSO4·7H2O. The tray was placed on a heated shaker at 28°C and 50–75 rpm for aeration. After 2 days, the J2s were separated from unhatched eggs and concentrated to a final optimized concentration of 1,000 J2 mL^-1^. Two milliliters of J2 inoculum were added to each root system. RKN (*M. incognita*) females were harvested from roots of peppers (*Capsicum annuum*) cultivar PA136 2–4 months after inoculation. Eggs used to inoculate roots of soybean seedlings (*Glycine max*, cv. Williams 82) were extracted using a 1% NaOCl solution [44]. The concentration of the egg suspension was adjusted to 1500 eggs mL^-1^. Two milliliters of inoculum were added to each root system. The plants were then grown in the greenhouse for 35 days, for both the SCN and RKN experiments. Confirmation of infection in representative infected root samples was performed by the acid fuchsin staining procedure of [50].

### Isolation of *PAD4* homolog from *Arabidopsis thaliana*

#### First strand cDNA synthesis

Total RNA was extracted from *Arabidopsis thaliana* leaves using the RNeasy Mini Kit (Qiagen, USA) and used to synthesize first-strand cDNA using the SuperScript III First-Strand Synthesis System for RT-PCR (Invitrogen, Carlsbad, CA)with oligo d(T)as primer according to the manufacturer’s instructions.

### Amplification and purification of *AtPAD4* cDNA

The Arabidopsis *PAD4* gene (accession No. NM_115103) was amplified from cDNA from *A. thaliana* leaves using gene specific primers (Table 4) to yield a 1626-bp product. We added a CACC sequence to the 5′ end of forward primer to enable insertion of the amplicon into the pENTR vector (Invitrogen). The PCR product was purified using the E-Gel® Electrophoresis System (Invitrogen).

### Gene cloning

The pENTR™ Directional TOPO Cloning Kit (Invitrogen, Carlsbad, CA) was used to clone *AtPAD4* into the pENTR cloning vector. The resulting construct was transformed into competent *Escherichia coli* cells using One Shot Mach1™ T1R chemically competent *E. coli* (Invitrogen, Carlsbad, CA), and the plasmid was harvested using the QIAprep Spin Miniprep Kit (Qiagen, Valencia, CA). Presence and orientation of *AtPAD4* were confirmed by DNA sequencing the positive samples using a 3130XL Genetic Analyzer (Applied Biosystems, Foster City, CA). *AtPAD4* was moved from the pENTR vector to the plant overexpression vector pRAP15 (Figure 1) using Invitrogen’s Gateway technology. The pRAP15 vector has a tetracycline resistance gene (TetR)for bacterial selection engineered into a BstEII site that lies outside the left and right borders and the enhanced green fluorescent protein (*eGFP*) gene [51]driven by the rolD root promoter [52,53]for visual screening of transformed roots [51]. The inserted *AtPAD4* gene was driven by the figwort mosaic virus subgenomic transcript (FMV-sgt) promoter [51], which exhibits strong, constitutive root expression. The cloning reaction was mediated by the Gateway LR Clonase™ II Enzyme Mix (Invitrogen,Carlsbad, CA)and involves crossing over between *att*R sites on pRAP15 and *att*L sites on pENTR, with *AtPAD4* replacing the lethal *ccd*B gene that is used for bacterial selection. Colony PCR was used to confirm the presence and/or orientation of *AtPAD4* and *eGFP* using the following primers sets: (1) FMV-F + *PAD4*-R; (2) *eGFP*-F + *eGFP*-R (Table 4).

### *Agrobacterium* transformation

The pRAP15 clone was moved into competent *Agrobacterium rhizogenes* K599 following the procedure of [54]. Plates were grown for 3 days at 30°C, and colonies were transferred to tubes of 5 mL TB liquid media containing 5 mg mL^-1^ tetracycline and incubated overnight at 37°C. Transformations were confirmed by PCR as described above.

### Plant transformation and challenge with *M. incognita* and *H. glycines*

A culture of *A. rhizogenes* transformed with either the empty pRAP15 control or pRAP15+ *RFP* and pRAP15+*AtPAD4* genes was grown in 5 mL TB liquid medium containing 5 mg mL^-1^ tetracycline overnight at room temperature on a rotary shaker at 250 rpm. The 5-mL culture was used to inoculate 600 mL of the same medium and then incubated under the same conditions. The culture was centrifuged at 5000 rpm at 4°C for 30 min. The pellet was resuspended in Murashige and Skoog medium (MS) with 3% sucrose. One hundred plants of a soybean cultivar (*G. max* cv. ‘William 82’) susceptible to parasitism by SCN and RKN were grown for each experiment for 9 days before transformation. Composite plants with roots transformed by *A. rhizogenes* were produced following the method of [51]. In brief, shoots were cut at the soil line, placed in the a suspension of *A. rhizogenes* in MS medium, vacuum infiltrated for 30 min, then incubated overnight at 23°C at 65 rpm in a were planted in 50-cell flats filled with pre-wetted Promix. MS medium inoculated with the same amount of water instead of the transformed *A. rhizogenes* culture was used in a mock transformation to produce non-transformed control (NC) plants. After incubation, the stems were rinsed with water, placed in a beaker of water, and incubated for approximately 48 hr at 23 ^0^C in a growth chamber. The plantlets were planted in pre-wetted Promix in the greenhouse. Four weeks after planting, the plants were screened to identify transformed roots using a Dark Reader Spot lamp (Clare Chemical Research, Dolores, CO).

For each experiment, 33 plants with the healthiest roots and strongest eGFP expression were selected 28 days after planting, and nontransformed roots were partially removed. The plants were replanted in soil and grown for an additional 14 days, after which 10 plants were selected for nematode assay, and all nontransformed roots were removed. Roots were challenged with 2000 *H. glycines* J2 per plant or 3000 *M. incognita* eggs per plant. Inocula were pipetted into 2 holes in the soil near the plant stem and about one inch deep. Ten plants were used for each experiment. Mature SCN females and RKN galls were counted 35 days after plant inoculation (dai).

### RKN galls counts and measurements

Thirty five days after inoculation with RKN, ten plants were uprooted. The roots were washed free of soil and fresh weights were recorded. RKN galls were counted. The sizes of ten RKN galls and the sizes of ten RKN nematodes within roots were determined by cutting the roots into 1 to 2-cm-pieces and staining with acid fuchsin according to [50]. Galls were placed on slides containing a drop of glycerol. No coverslip was used. The area of each gall and RKN was determined by circumscribing the profile of each using the laser capture microscope Leica Microsystem platform and software version 5.0. The same magnification was used for all samples.

### SCN female counts

Mature SCN females were collected from individual plants over nested 20- and 60-mesh sieves. Collected females in ~30 mL of water were washed into 150 ml beakers. The females were poured onto 9-cm diameter filter paper (Schleicher and Schuell; Keene, NH) in a Buchner funnel system under constant vacuum. Counting was done under a dissecting microscope. Both the RKN and SCN experiments were analyzed by *t*-test using the GraphPad software (La Jolla, CA).

### Confirmation of the effectiveness of the plant overexpression vector

The functionality of the plant overexpression vector pRAP15 was confirmed by using it to overexpress a red fluorescent protein (RFP) in soybean roots. The pRAP15+*RFP* construct was cloned as described above at USDA-ARS, Soybean Genomic and Improvement Laboratory, Beltsville, MD, USA, using the primers *RFP*-F and *RFP*-R (Table 1). The vector, pRAP15 contains the figwort mosaic virus subgenomic transcript (FMV-sgt) promoter driving the expression of the tandem inverted repeat cassette. This promoter exhibits strong, constitutive root expression throughout the entire course of *H. glycines* infection. Images of roots expression of eGFP and RFP were obtained using a Zeiss 710 Laser Scanning Confocal Microscope (LSCM) and a Zeiss Axio Observer™ inverted microscope with a 40x1.2 NA water immersion plan apochromatic objective. An Argon laser was used to excite eGFP at 488 nm and emission was monitored between 500 to 510 nm with a MBS 488/561/633 filter set. RFP was excited at 561 nm with a diode pumped solid state laser and the emission detected at 575 to 620 nm with the 488/561/633 filter set. Zeiss ZenTM 2009 was used to capture the images and Axiophot 4.6™ and Photoshop 7.0™ were utilized to design the figures.

### Molecular analysis of putative transgenic plants

Genomic DNA isolated from roots of healthiest transgenic roots that displaying the strongest *eGFP* fluorescence and control soybean plants using a DNeasy plant mini kit (Qiagen, USA). The presence of the *AtPAD4* gene in the transgenic roots was confirmed by PCR.

Sequence specific primers (Table 1) were used for amplification the *AtPAD4* DNA fragment using plant genomic DNA as template. DNA extracted from untransformed plants was used as negative control. Also, primers amplifying fragments of approximately 132 bp from the soybean ubiquitin-3 gene, GenBank accession D28123, were used to confirm that soybean DNA was present in all samples. The PCR conditions included initial melting temperature of 94°C for 2 min followed by 35 cycles of 94°C for 30 s, 65°C for 30 s and 72°C for 2 min. This was followed by a final extension time of 10 min at 72°C. The PCR mixture included 0.4 μl *Taq* polymerase (Invitrogen, Carlsbad, CA, USA), 50 mM Mgcl2 and 10 mM dNTPs. The template plasmid DNA concentration was 1–10 ng μl^-1^. The amplified PCR fragments were resolved on a 0.8 g/ml agarose gel and observed under ultraviolet light.

### Quantitative real-time reverse transcription polymerase chain reaction (qRT-PCR) to determine the transcript level of *AtPAD4 and* defense genes inside plant roots

RNA was extracted from three individual roots (100 mg each), roots transformed with the empty pRAP15 (as a control) having the strongest *eGFP* expression and representing independent transformation events using the Ultra Clean Plant RNA Isolation Kit (MOBIO, Carlsbad, CA). The RNA was treated with DNase I to remove genomic DNA. The RNA was utilized to synthesize single-stranded cDNA using reverse transcriptase (Invitrogen, Carlsbad, CA) and oligo dT primers, according to the manufacturer’s instructions. All the primer sets were designed to flank a region that contains one intron to make sure that the expected size product was amplified from cDNA and not from genomic DNA. Primers (Table 2) were designed to be specific to the flanking region of the Arabidopsis *PAD4* (*AtPAD4*) and to yield PCR-amplified fragments of approximately 150 bp. Also, the soybean ubiquitin-3 gene, GenBank accession D28123 used as a positive RT-PCR control for the experiment to confirm that the soybean RNA was present in all samples. And the soybean genes (*GmPAD4*; *GmEDS1*) Phytozome accession Glyma06g16290.1; Glyma06g19920.1 and (*GmPR1*) GenBank accession XM_003545723.1as related defense genes.

Other controls for qRT-PCR included reactions containing no template and qRT-PCR reactions containing no reverse transcriptase. qRT-PCR was performed on three biological replicates and each reaction was replicated three times. Relative quantities of gene expression were determined using the Stratagene Mx3000P Real-Time PCR system (Stratagene, La Jolla, CA) as described by the manufacturer. DNA accumulation during thereaction was measured with SYBR Green. The Ct (cycle at which there is the first clearly detectable increases in fluorescence) values were calculated using software supplied with the Stratagene Mx3000P Real-Time PCR system. SYBR green dissociation curve of amplified products demonstrated the production of only one product per reaction. Data analysis was performed according to the sigmoidal model described by [31] to get absolute quantification. The PCR products were run on 0.8% agarose gel and visualized under UV light.

## Authors’ contributions

BM carried out the design of the experiments, designed the overexpression vector, helped to draft the manuscript and performed the statistical analysis. RY carried out the molecular genetics and nematodes studies, transformation, performed the statistical analysis and drafted the manuscript. KK participated in the design and construction of the overexpression vector. MM participated in the nematodes. EB participated in the statistical analysis and helped to draft the manuscript. GB conducted the Laser Scanning Confocal Microscope studies. All authors read and approved the final manuscript.
